# Comprehensive telemedicine solution for remote monitoring of Parkinson’s disease patients with orthostatic hypotension during COVID-19 pandemic

**DOI:** 10.1007/s10072-022-05972-6

**Published:** 2022-03-17

**Authors:** Paola Polverino, Miloš Ajčević, Mauro Catalan, Claudio Bertolotti, Giovanni Furlanis, Alessandro Marsich, Alex Buoite Stella, Agostino Accardo, Paolo Manganotti

**Affiliations:** 1 Clinical Unit of Neurology, Department of Medicine, Surgery and Health Sciences, University Hospital and Health Services of Trieste - ASUGI, University of Trieste, Strada di Fiume, 447–34149 Trieste, Italy; 2 Department of Engineering and Architecture, University of Trieste, Via A. Valerio, 10-34127 Trieste, Italy; 3 Televita, Piazza San Giovanni, 6-34122 Trieste, Italy

**Keywords:** e-Health, Parkinson’s disease, Autonomic dysfunction, Telemonitoring, COVID-19

## Abstract

**Objective:**

Orthostatic hypotension (OH) represents a frequent but under-recognized phenomenon in Parkinson’s disease (PD). During COVID-19 pandemic, Information and Communication Technologies (ICT) have become pivotal in the management of chronic diseases like PD, not only to assess motor impairment, but also for vital signs monitoring. This pilot study aimed to propose a real-time remote home-monitoring system and protocol for PD patients with OH.

**Methods:**

Vital parameters were acquired by wireless devices and transmitted to an ICT platform, providing data and smart notifications to the healthcare provider through an interactive web portal. Eight patients with idiopathic PD and OH underwent 5-day monitoring. Data about OH episodes, therapeutic interventions, impact on daily activities, and patient satisfaction were collected and analyzed.

**Results:**

The proposed solution allowed the identification of 65 OH episodes and subsequent medical interventions. Thirty-five episodes were asymptomatic, especially in the postprandial and in the afternoon recordings. Systolic-blood-pressure (SBP) and diastolic-blood-pressure (DBP) were significantly lower in symptomatic episodes, while the pressure drops resulted significantly higher in presence of symptoms. High usability and patient satisfaction scores were observed.

**Conclusion:**

The proposed home-monitoring system and protocol have proved to provide useful information and to allow prompt interventions in the management of PD patients with OH during COVID-19 pandemic.

## Introduction

Among non-motor features of Parkinson’s disease (PD), orthostatic hypotension (OH) represents a frequent yet overlooked phenomenon, showing a prevalence of 30–60% in PD population [1]. This symptom results from the impairment of baroreflex mechanisms necessary to maintain a constant blood pressure (BP) and cerebral perfusion across supine, sitting, and standing positions.

A degree of damage to the postganglionic sympathetic system is suggested as a possible pathophysiologic mechanisms of OH in PD, including noradrenergic denervation in both cardiac and extracardiac regions and arterial baroreflex failure [2]. OH is defined by a BP fall greater than 20 mmHg of systolic blood pressure (SBP) and/or 10 mmHg of diastolic blood pressure (DBP) within 3 min of standing from a sitting or supine position [3]. Heart rate should normally show an increase of 4–6 beats per minute upon standing, with a greater increase in case of OH; in presence of autonomic dysfunction, this physiologic response is usually impaired [4].

An accurate blood pressure assessment should always be performed as part of the clinical evaluation of PD patients because of the presence of both OH together with the phenomenon of supine hypertension [5], which is an inter-related aspect of OH that could be worsened by treatments used to manage OH itself [6, 7]. Isolated measurements of SBP do not always reflect the presence of symptomatic OH, due to the possible circadian fluctuations in blood pressure values. A 24-h ambulatory BP monitoring or telemedicine connected BP monitoring systems can be extremely useful in the assessment of OH in Parkinson’s disease, in order to better evaluate the correlation between symptoms and blood pressure and to identify potentially dangerous asymptomatic BP drops [8]. Furthermore, as aforementioned, the information about HR increase as well as a comprehensive Autonomous Nervous System (ANS) assessment may add useful information to the clinical evaluation. Comprehensive pathology and patient-specific e-Health solutions are necessary to allow the better management of chronic patients, as well as to improve psychological aspects [9].

Since the global spread of severe acute respiratory syndrome coronavirus 2 (SARS-CoV-2), the ongoing COVID-19 pandemic induced the reduced movement modality of the citizens [10, 11]. Continuous advancement of information and communication technologies (ICT) and wearable electronic devices allowed a remote patient monitoring, including the main vital signs. Remote patient monitoring can be even more useful to support physicians in extraordinary situations such as the COVID-19 emergency scenario [12, 13] that affected the way patients access the healthcare system [14–16]. In this context, a system of home telemonitoring of vital parameters (BP, HR, oxygen saturation (SpO_2_), and body temperature) could play a fundamental role in the care setting of PD patients complicated by the presence of OH.

There is a growing research interest in the assessment of PD motor aspects using telemedicine devices [17–19]. On the other hand, dysautonomia and in particular BP telemonitoring results a less investigated issue with less available data in PD subjects. Furthermore, only limited information about the OH symptom burden on activities of daily living has been reported. In this context, the majority of studies reported vital signs recordings performed in the hospital setting or using 24-h ambulatory BP monitoring (ABPM) [20]. These systems allow frequent measurements, but cannot be reviewed in real time and, thus, are unable to ensure patient’s safety or protocol compliance [21].

The aim of this study was to propose a real-time remote home-monitoring system and protocol for patients with Parkinson’s disease and orthostatic hypotension, as well as to preliminary assess its potential utility. The established endpoints were to assess OH episode frequency and severity, to investigate symptom burden and its impact on the activities of daily living and to describe the provided measures for neurogenic orthostatic hypotension (nOH) management. In addition, the usability and patients’ satisfaction of the proposed e-Health system were investigated through the administration of a specific questionnaire.

## Materials and methods

**Fig. 1:**
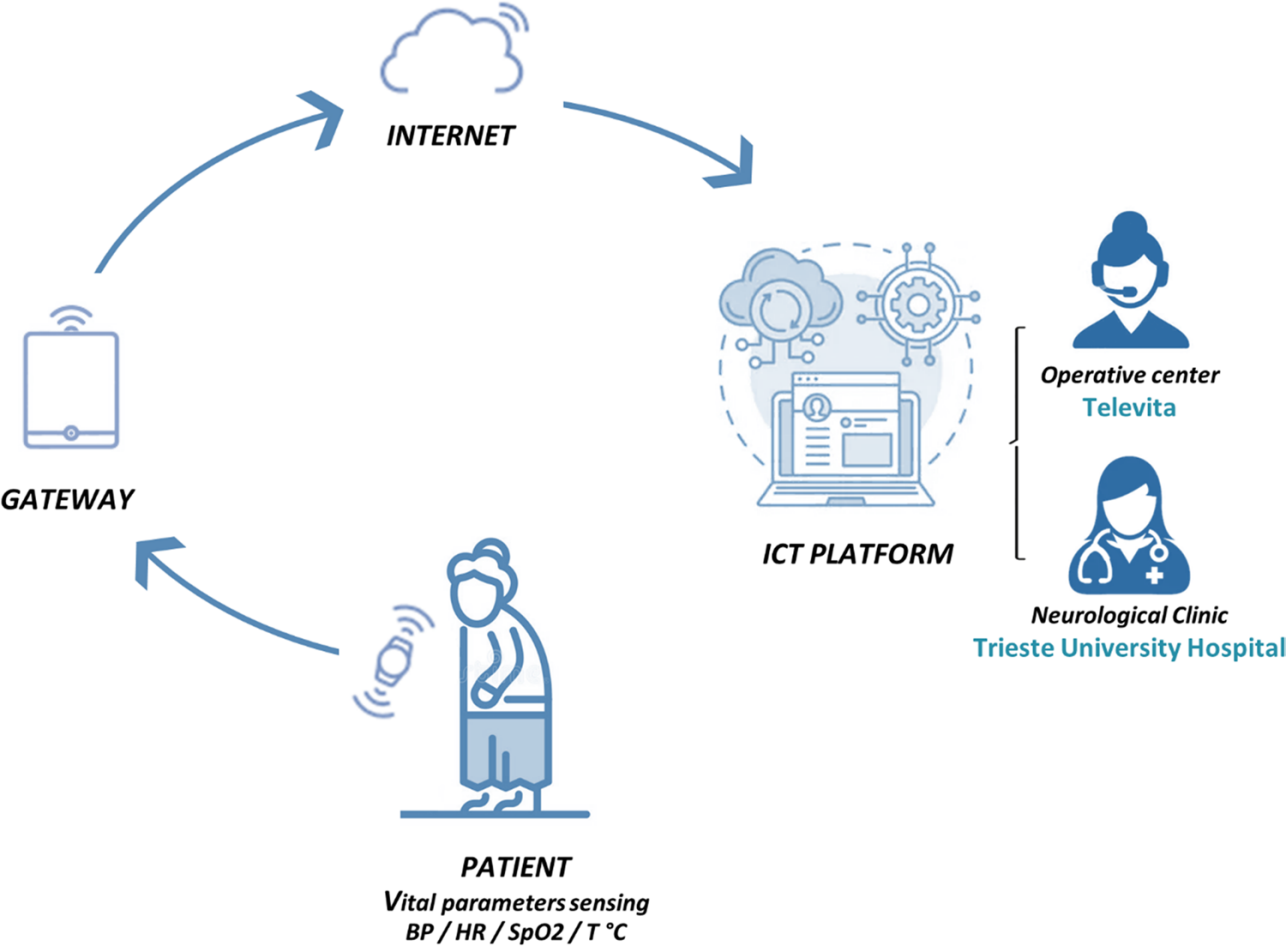
Block diagram of the proposed remote home-monitoring system and protocol for PD patients with orthostatic hypotension during COVID-19 pandemic

The schematic diagram of the proposed telemonitoring system, consisting of portable vital signs sensing devices (BP, HR, SpO_2_, and temperature), panic button, getaway, and a dedicated ICT platform, is reported in Fig. 1.

Vital parameter data were acquired by wireless portable devices. The data were transmitted throughout the gateway to the ICT platform using mobile broadband connectivity, thus, providing raw and processed data and notifications to the Televita Operative Centre and the Neurological Clinic of Trieste University Hospital. In particular, blood pressure was recorded by using BP monitoring device (Diamond Cuff BP - P80, ForaCare), blood oxygen level was acquired by pulse oximeter (OxyWatch, ChoiceMMed), and heart rate (HR) by using both OxyWatch and Diamond Cuff P80 while body temperature was measured by (FORA IR21, ForaCare) device.

A dedicated interactive web-portal, which also provided smart-alarms, allowed patients and caregivers to review data in real-time and to establish communication with operators or physicians if needed. Vital signs measurements were performed 3 times a day (at morning after awakening, after meal, and in the afternoon), as well as on patient’s demand in case of relevant OH symptoms. Patients were instructed to take each BP and HR measurement both in supine position (after a supine rest of at least 5 min) and after 3 min of standing. OH episodes were automatically identified if SBP fall was greater than 20 mmHg and/or if DBP fall was greater than 10 mmHg within 3 min of standing from a sitting or supine position. Three different severity thresholds for all vital parameters were established. In case of thresholds exceeding, a dedicated operator checked measurement reliability, verifying by phone contact if vital signs recording was performed correctly by the patient. After this step, an alarm was set in order to alert the physician, so that he could visualize in real-time all measurements, establish a phone consulting, and provide the appropriate intervention according to clinical severity. Patients were phone-interviewed about the clinical status, and symptoms suggestive of OH were collected. Furthermore, patients were asked to quantify the global impact of nOH symptoms on their daily life activities, according to a self-rating scale of severity ranging from none to very severe.

In order to evaluate the proposed solution and preliminary assess its potential utility, we prospectively enrolled 8 patients afferent to the Movement Disorder Unit of our Centre in a period between June 2020 and July 2020. Inclusion criteria included a diagnosis of idiopathic PD with a formal diagnosis of OH or suggestive symptoms of OH. Patient data such as age, sex, disease duration, LEDD, Levodopa intake schedule, and years experiencing OH symptoms were collected. Selected patients were screened for comorbidities and exclusion criteria were the presence of diabetes mellitus, other diseases potentially associated with autonomic neuropathy, or medical conditions associated with non-neurogenic OH. Each patient underwent a monitoring of vital signs (blood pressure, heart rate, oxygen saturation, and temperature) for a period of 5 days. At the end of the monitoring period, the Telehealth Usability Questionnaire (TUQ) [22] was administered to each participant to evaluate the usability of the proposed e-Health solution. The TUQ was designed to be a comprehensive questionnaire covering different items regarding usability factors, such as usefulness, ease of use, effectiveness, reliability, and satisfaction. Each item was assessed through different questions, each of them answered with a score ranging from 1 to 7, so that the mean scores of the different items were calculated.

Statistical analysis was carried out by using SPSS Statistics 21.0 (IBM, Armonk/NY, USA). Kolmogorov–Smirnov test was used to evaluate the normal distribution of variables. Continuous variables with a normal distribution are presented as mean ± SD, those with a skewed distribution as median and interquartile ranges (IQRs), and categorical variables as counts and percentages (%).

Measurements of HR, SBP, and DBP were collected for all patients in each day period. SBP and DBP clinostatism-orthostatism drops were then calculated for every collected measurement, allowing the identification of OH episodes. In addition, mean ± SD values of all BP and HR measurements in supine and standing positions were calculated for each day period, as well as mean ± SD values of SBP and DBP drops and HR increases considering only OH episodes. SBP and DBP drops and HR increases recorded during the identified OH episodes were compared among the three different day periods. Differences between symptomatic and asymptomatic episodes were also investigated.

## Results

Clinical and demographic characteristics of included patients are reported in Table 1. All patients experienced symptoms suggestive of OH in past, and the majority of them (6 out of 8) received a previous formal diagnosis. Three out of 8 patients had also a diagnosis of a concomitant hypertension, treated with antihypertensive drugs. During the study period, all patients were receiving dopaminergic therapy.

**Table 1:** Patients’ clinical and demographic characteristics

	Patients (*N* = 8)
*Age (y)*	77.87 ± 5.22
*Sex (M/F)*	5/3
*Disease duration (y)*	8 ± 5.61
*LEDD*	477.75 ± 256.69
*Years experiencing nOH symptoms*	4.00 ± 2.78
*< 1*	0/8
*1–4*	6/8
*5–9*	1/8
*≥ 10*	1/8
*Formal OH diagnosis*	6/8
*Diagnosis of hypertension*	3/8

*y*, years; *LEDD*, levodopa equivalent daily dosage; *nOH*, neurogenic orthostatic hypotension; *OH*, orthostatic hypotension

The proposed e-Health solution allowed the identification of 65 OH episodes and subsequent medical interventions. OH episodes were identified in all included patients, including two cases without a previous formal diagnosis.

OH episodes were distributed as follows: 21 episodes (32.3%) in the morning, 25 (38.5%) in the postprandial period, and 19 (29.2%) in the afternoon. In Table 2 are reported mean ± SD values of all performed measurements of the blood pressures and heart rates in supine and standing positions for each day period, as well as mean ± SD values of pressure drops and heart rates observed during identified OH episodes. Baseline supine–measured SBP and DBP were significantly higher in the morning compared to those measured in postprandial (*p* < 0.001) and afternoon (*p* < 0.001) periods. During identified OH episodes, a slightly higher, although non-significant, DBP drop was observed in the morning compared to postprandial and afternoon periods. No significant differences in the identified SBP drops among the three different day periods were detected.

**Table 2:** Mean ± SD values of all performed measurements of the blood pressures and heart rates in supine and standing positions, as well as mean ± SD values of pressure drops and heart rates observed during identified OH episodes

	Morning (supine)	Morning (standing)	Postprandial (supine)	Postprandial (standing)	Afternoon (supine)	Afternoon (standing)
*All measurements*						
SBP	145.59 ± 17.54^*^	120.26 ± 30.96	127.05 ± 20.53	108.80 ± 21.32	129.20 ± 14.12	116.92 ± 25.26
DBP	91.78 ± 13.95^*^	79.31 ± 15.12	79.35 ± 13.43	70.15 ± 14.21	79.80 ± 10.60	75.25 ± 15.46
HR	68.43 ± 9.83	76.31 ± 11.06	72.42 ± 10.98	80.85 ± 14.03	69.70 ± 9.20	78.40 ± 11.89
*OH episodes*						
SBP drop	-	46.85 ± 22.36	-	42.42 ± 14.22	-	37.19 ± 14.18
DBP drop	-	34.07 ± 19.03	-	22.87 ± 10.62	-	22.44 ± 10.13
HR increase	-	6.37 ± 6.09	-	4.09 ± 3.52	-	6.94 ± 4.37

*SBP*, systolic blood pressure expressed in (mmHg); *DBP*, diastolic blood pressure expressed in (mmHg); *HR*, heart rate expressed in (bpm). * significantly different compared to post-prandial (*p* < 0.001) and afternoon (*p* < 0.001) supine measurements

HR evaluation showed that in 57 out of 65 episodes of OH (87.7%), there was a concomitant impairment of the chronotropic compensatory response with an increase of HR lower than 15 bpm. No alterations of temperature or SpO_2_ parameters were observed.

**Fig. 2:**
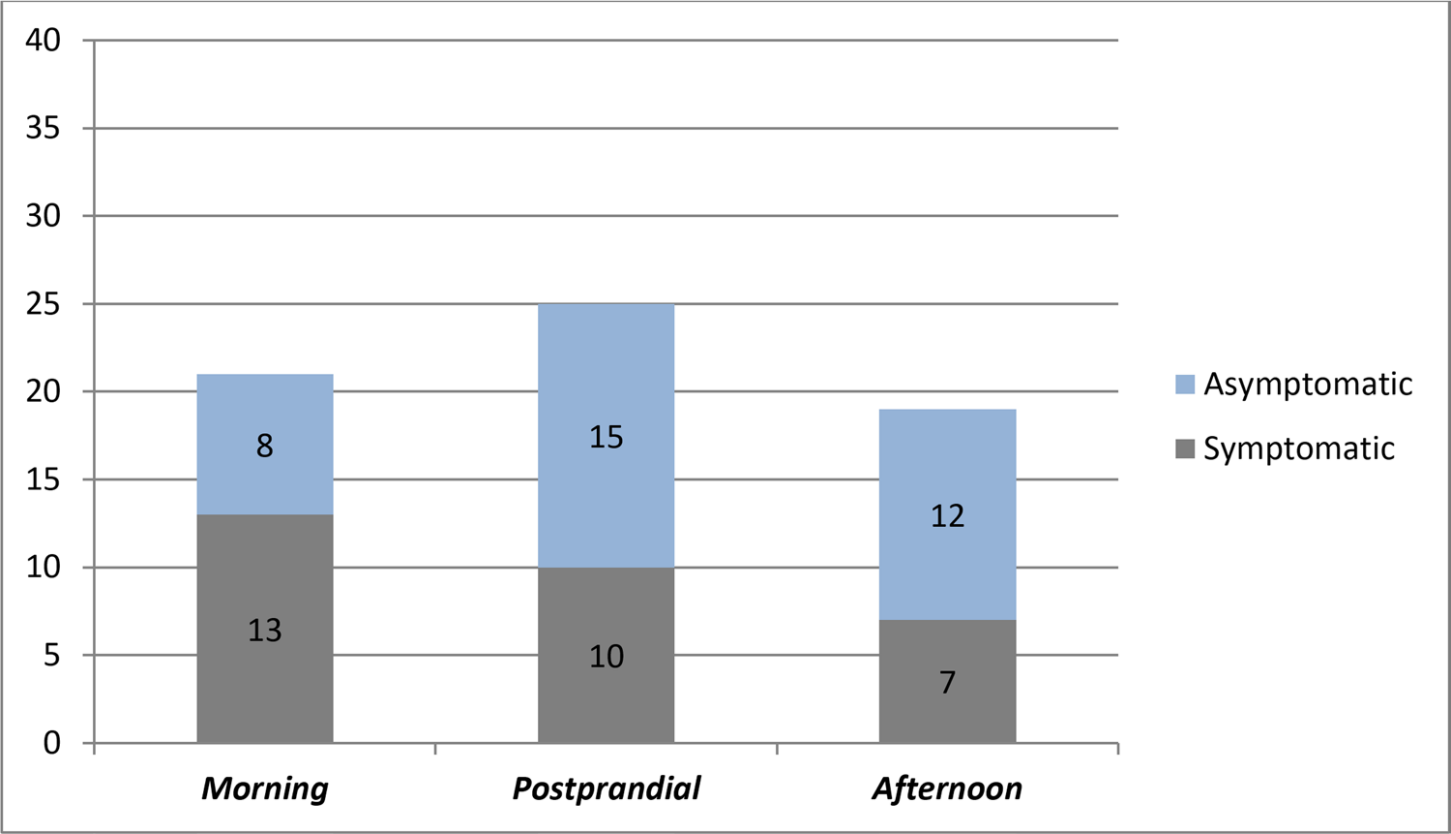
Number of asymptomatic and symptomatic OH episodes during the three different daily measurements

Asymptomatic and symptomatic OH episode distribution is reported in Fig. 2. Thirty-five out of 65 (53.8%) identified OH episodes were reported as asymptomatic, with a higher frequency in the postprandial (15/25; 60%) and in the afternoon (12/19; 63.2%) recordings versus morning measurements (8/21; 38.1%), which resulted in most cases symptomatic. No significant difference in frequency of symptomatic episodes was observed among three reference periods.

Mean ± SD values of SBP and DBP in clinostatism and orthostatism, as well as of SBP and DBP drops during symptomatic versus asymptomatic OH episodes, are reported in Table 3. In orthostatism, SBP and DBP were significantly lower in symptomatic episodes (*p* < 0.001 and *p* = 0.005 respectively), while SBP and DBP drops resulted significantly higher in presence of symptoms (*p* < 0.001 and *p* = 0.001 respectively).

**Table 3:** Mean ± SD of SBP and DBP in clinostatism and orthostatism, as well as of SBP and DBP clinostatism-orthostatism drops during symptomatic versus asymptomatic OH episodes

	Symptomatic	Asymptomatic	*p-value*
*Clinostatism*			
SBP	138.90 ± 20.72	132.57 ± 14.95	0.159
DBP	90.67 ± 16.65	83.54 ± 12.52	0.054
*Orthostatism*			
SBP	89.87 ± 11.45	107.00 ± 15.13	< 0.001
DBP	62.03 ± 9.78	69.91 ± 11.77	0.005
*BP drop (Clin-Orth)*			
SBP drop	49.10 ± 18.02	25.86 ± 19.11	< 0.001
DBP drop	28.67 ± 15.78	16.23 ± 11.93	0.001

*SBP*, systolic blood pressure; *DBP*, diastolic blood pressure. All BP values are expressed in mmHg

**Fig. 3:**
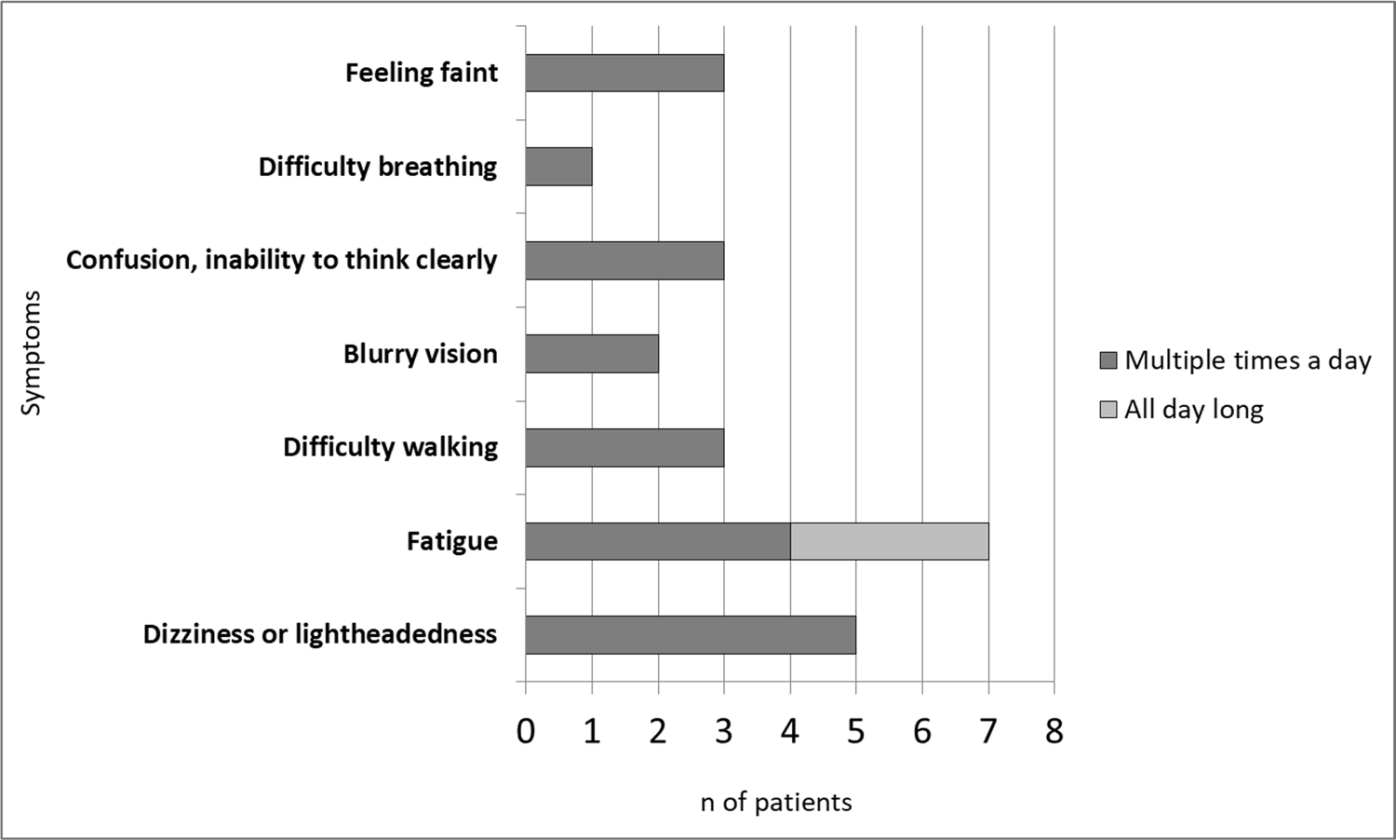
Number of patients reporting different neurogenic orthostatic hypotension (nOH) symptoms

The most frequently reported symptoms of nOH were dizziness or lightheadedness, and fatigue when standing, followed by feeling faint, confusion, and difficulty walking (Fig. 3). Symptoms were reported as constantly present or occurring multiple times a day, triggered by positional changes from sit to standing or when patients were standing for longer periods of time. Other less reported symptoms were blurry vision and difficulty breathing. Falls caused by OH symptoms (at least one in the previous year) were reported by 3 out of 8 patients (37.5%) with a mean value of 2.7 falls.

**Fig. 4:**
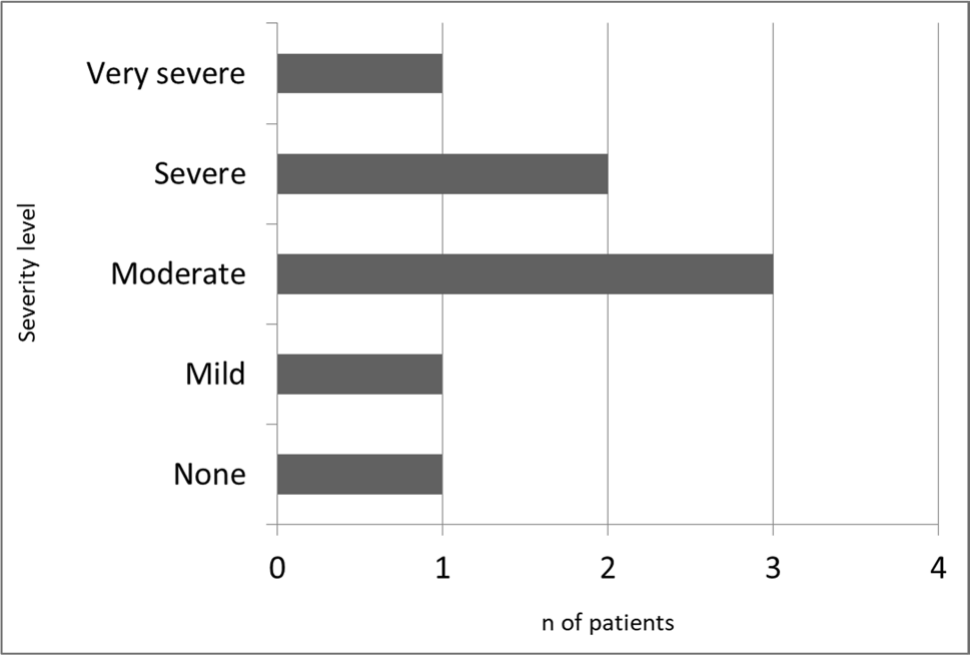
Self-reported impact of neurogenic orthostatic hypotension (nOH) symptoms on daily activities

A total number of 44 medical interventions were performed by a dedicated neurologist using the phone teleconsulting, in order to provide the appropriate recommendations for symptom management. In particular, the following non-pharmacological measures were suggested: avoid quick positional changes, increase fluid intake, compression stockings, avoid heated environments, night time head-up tilt, and avoid alcohol intake and carbohydrate-heavy meals. No adjusting/discontinuation of blood pressure/heart medications or dopaminergic treatment was applied, neither pharmacological treatment for OH was introduced. Patients were asked to quantify the global impact of nOH symptoms on their daily life activities, according to a self-rating scale of severity ranging from none to very severe. As shown in Fig. 4, the majority of patients (7/8) reported a global negative impact of OH on daily life activities and functional independence; most patients self-rated the global impact as moderate (3/8) and severe (2/8).

**Fig. 5:**
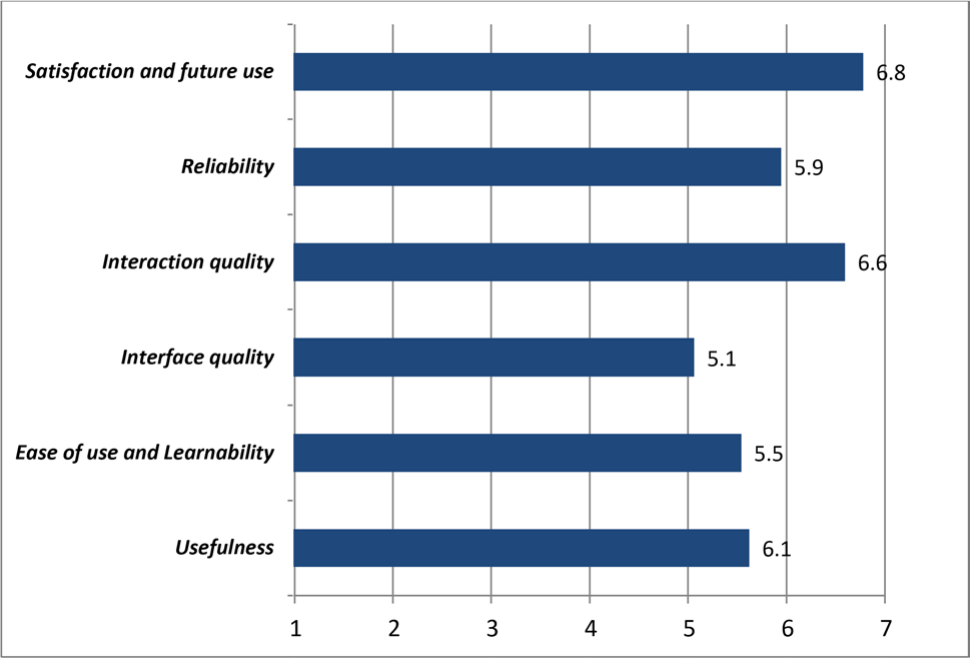
Evaluation of the usability of the proposed e-Health solution. Average scores for each domain of the TUQ (Telehealth Usability Questionnaire) (scores: min: 1 - max: 7)

In Fig. 5, average scores of different items of TUQ questionnaire are reported. High usability and patient’s satisfaction scores were observed.

## Discussion

The novelty of this pilot study is the preliminary-assessed utility of the proposed comprehensive telemedicine solution for real-time home monitoring of vital signs in patients with Parkinson’s disease and orthostatic hypotension. Compared to other studies [20, 23], using home BP holter recordings, a remote real-time monitoring system, could increase patient’s safety and compliance, as well as data reliability.

According to the results of this pilot study, the application of the proposed telemonitoring system in this particular subset of patients reveals relevant information with crucial clinical and health care management implications. e-Health solutions for PD patients so far have been mostly applied to investigate PD motor aspects [19, 24, 25], such as ON-OFF fluctuation, postural stability, and gait disturbances, but only few studies have focused on real-time monitoring of autonomic impairment [21].

COVID-19 pandemic increased dramatically the need for telemonitoring systems in order to manage chronically ill patients during lockdown period [26] and created the opportunity to extend the fields of applications of already available devices on different pathologic conditions, as we performed in our experience.

OH represents a major source of disability and an independent risk factor for mortality in elderly and in the PD population, leading to postural instability, gait impairment, and repetitive falls with consequent increase of accesses to Emergency Room (ER) for head trauma and fractures [27]. An early detection and a correct management of this dysautonomic complication are thus of paramount importance in reducing the need for access to hospital facilities. Noteworthy, 2 out of 8 patients in our study were reporting symptoms suggestive of OH, without a formal diagnosis; in these patients, the telemonitoring system allowed the clinician to detect OH episodes and associated symptoms and to perform further ANS testing to confirm the OH diagnosis. The management of OH in PD population is sometimes very challenging, considering also the associated hypotensive effect of dopaminergic drugs, increasing the overall autonomic symptoms burden [28]. OH can occur with or without symptoms, so despite its frequency, this phenomenon is frequently under-recognized. We found that thirty-five out of 65 (53.8%) OH episodes were reported as asymptomatic, with a higher rate in the postprandial (60%) and in the afternoon (63.2%) recordings compared to morning measurements (38.1%) that were mostly accompanied by symptoms. Moreover, the lower absolute SBP and DBP mean values (both in supine and standing positions) were recorded during postprandial and afternoon measurements, while higher absolute values of both SBP and DBP were found in the morning after awakening. These findings may be due to the physiological circadian rhythm involving the activation of sympathetic nervous system and to a not negligible impact of morning hypertension in 3 of our patients.

As a main finding, our analysis comparing symptomatic versus asymptomatic episodes showed lower SBP and DBP mean values (in standing position) together with higher SBP and DBP drops when symptoms were present.

These data are in agreement with literature sustaining that the presence of symptoms seems to depend mostly on absolute BP value in the standing position rather than on the magnitude of BP drop [29, 30].

Supporting this notion, a study with 210 patients with PD reported that standing mean BP < 75 mmHg predicted presence of OH symptoms with a sensitivity of 97% and a specificity of 98%. On the other hand, only 31% of patients with OH reported OH symptoms and these diagnostic criteria predicted presence of symptoms with a sensitivity of 92% and a specificity of 58% [30].

Moreover, another study with 205 patients with exaggerated SBP reduction (> 60 mmHg) during head up tilt test suggested that 33% of patients were asymptomatic, whereas the magnitude of SBP drop was similar in symptomatic and asymptomatic patients [31]. On the contrary, our results showed the presence of a significant difference of both systolic and diastolic blood pressure drops in symptomatic versus asymptomatic OH episodes, suggesting that also the magnitude of BP drop could affect the presence of orthostatic symptoms. These data highlights the crucial clinical implications of using a home telemonitoring system in PD patients with dysautonomia to improve OH detection and management, especially taking into account the high rate of totally asymptomatic episodes and their potential clinical complications.

Patients with PD represent a higher risk category for OH, considering all the other possible concomitant risk factors such as medication use, comorbidities, reduced physical activity, and dehydration. This scenario is mandatory to consider the prognostic role of OH: about this topic, numerous large prospective studies have demonstrated a relationship between OH and adverse cardiovascular (CV) and non-CV outcomes such as cognitive decline, decreased physical functioning, risk of falls, and late-life depression, confirming the importance of an early detection of this phenomena, even when asymptomatic, in this at risk population [30].

In our study, 65 total episodes of OH were recorded during the whole monitoring. OH episodes occurred in a higher rate after meal (38.5%) and in the morning (32.3%), followed by afternoon recordings (29.2%).

Orthostatic hypotension is believed to occur more frequently in the morning after awakening and after large meals (postprandial OH). From a pathophysiological point of view, volume depletion due to increased nocturnal diuresis induced by supine hypertension is the proposed mechanism at the bases of morning OH episodes [32]. On the other hand, the venous pooling occurring after large meals is probably responsible of postprandial OH, together with a possible contribution of insulin-mediated vasodilatation [30]. According to our findings, a BP monitoring focusing on potential situations of orthostatic stress (such as early hours in the morning after awakening) or possible triggers (meals, physical activity) is crucial to increase the possibility to capture OH episodes which may remain otherwise silent and unrecognized, providing an accurate assessment and management of nOH phenomena.

Furthermore, HR has also been assessed in our patients, showing that in 87.7% of OH episodes was observed a concomitant increase of HR lower than 15 bpm. Our findings lead support to the concept that HR evaluation is important to perform in the context of nOH assessment, since a failure of the chronotropic compensatory response (< 10–15 bpm) is supportive of autonomic impairment and may represent a practical tool to detect nOH [30]. As previously reported in the “Results” section, no alterations in temperature and oxygen saturation were recorded. The inclusion of these further parameters in the monitoring system might have important implications in the context of COVID-19 pandemic emergency, especially in a frailer population such as Parkinsonian patients and could also be useful to minimize confounding factors regarding OH clinical manifestation.

When symptoms were present, patients mostly reported a sensation of dizziness or lightheadedness and fatigue when standing followed by feeling faint, confusion, and difficulty walking. Symptoms presented as a constant complaint like in the case of fatigue or multiple times a day, triggered by positional changes from sit to standing or during longer periods of orthostatism.

The assessment and prompt management of symptoms are fundamental because symptomatic burden can lead to immobility and reduced level of functional independence, which in turn further worsen the severity of OH [33]. The impact extent of symptoms on daily life activities has been also quantified with a self-rating scale of severity, confirming the striking negative effect of OH on daily life activities and functional independence in the majority of patients. Interestingly, falls caused by OH symptoms (at least one in the previous year) were reported by 3 out of 8 patients (37.5%) in our sample, with a mean of 2.7 falls. These data suggest that the implementation of an e-Health system in this population may have a great impact on reduction of hospital admissions due to falls and related traumas.

On the other hand, the presence of OH symptoms alone may not represent an accurate indicator of tissue hypoperfusion especially in patients with PD. Symptoms of OH are frequently non-specific or sometimes indistinguishable from a levodopa “off” state and syncope can also occur with little or no premonitory symptoms in PD. Consequently, PD patients suffering OH are often untreated/undertreated, leading to greater disability and healthcare assistance need than patients without dysautonomic symptoms [34].

In these complex situations, a telemedicine-based monitoring allowed to shorten distances between physicians and patients, providing a real-time window on patients’ clinical status necessary to establish prompt interventions.

Our data showed that a phone teleconsulting was necessary in 44 occasions during the whole monitoring and appropriate non-pharmacological measures to overcome OH episodes were suggested. During teleconsulting, patients were asked about their clinical status and their complaints in correspondence of the OH episode detection; moreover, their compliance to the recommended strategies was also monitored, showing an overall satisfactory adherence to the provided recommendations with in some cases subjective relief from symptoms.

Although consensus guidelines for the pharmacologic treatment of OH are lacking, randomized placebo-controlled clinical trials show that effective treatment of OH can help reduce symptomatic burden [35], increase physical activity levels [36], and improve motor functionality [37]. This gives strength to the concept that a prompt recognition of OH and an adequate treatment may help prevent these negative consequences.

Finally, to assess the level of patient’s satisfaction about the proposed telemonitoring solution and service, the TUQ questionnaire was administered. The results obtained showed high average scores in all the explored items, even in the ease of use and learnability sub-item, pointing out a good usability of this telemedicine system also in the elderly. Interestingly, the higher average scores were recorded in the items of satisfaction and future use, reliability, and interaction quality, highlighting promising future perspectives and new potential fields of application of the proposed system.

In conclusion, this pilot study proposed a real-time remote home-monitoring system and protocol for patients with Parkinson’s disease and orthostatic hypotension and preliminary assessed its utility. The results highlighted the great usefulness of a PD–dedicated e-Health program able to monitor blood pressure, heart rate, SpO_2_, and temperature, in patients complicated with autonomic impairment manifesting OH, which might be further exacerbated by COVID-19 [38].

Despite some limitations due to the small sample size and the absence of a continuous monitoring, our data underline the clinical relevance of a prompt detection and management of OH phenomena in PD patients, in order to not underestimate this frequent complication and to reduce its consequences both on clinical status and on the need for health care assistance.

The proposed home-monitoring system and protocol have demonstrated to be feasible and to allow faster interventions in response to potential safety concerns in the PD population with OH, as well as in other pathologic conditions characterized by autonomic impairment. This could be particularly important in the PD population with OH, during the COVID-19 pandemic emergency period. Therefore, encouraging these alternative models of care based on the ICT technologies is of striking importance for the development of a patient-tailored care system with relevant implications on clinical management, research, and health care system organization. Moreover, the implemented video and phone teleconsulting has shown to be pivotal in this at risk population, especially during the emergency period, and may represent in synergy with vital signs smart-monitoring an effective and feasible strategy to provide the continuum of care and support health care system in the management of this potentially harmful PD complication.
